# Plant Polyphenols and Oxidative Metabolites of the Herbal Alkenylbenzene Methyleugenol Suppress Histone Deacetylase Activity in Human Colon Carcinoma Cells

**DOI:** 10.1155/2013/821082

**Published:** 2013-02-11

**Authors:** Isabel Anna Maria Groh, Chen Chen, Claudia Lüske, Alexander Thomas Cartus, Melanie Esselen

**Affiliations:** Division of Food Chemistry and Toxicology, Department of Chemistry, University of Kaiserslautern, Erwin-Schroedinger-Straße 52, 67663 Kaiserslautern, Germany

## Abstract

Evidence has been provided that diet and environmental factors directly influence epigenetic mechanisms associated with cancer development in humans. The inhibition of histone deacetylase (HDAC) activity and the disruption of the HDAC complex have been recognized as a potent strategy for cancer therapy and chemoprevention. In the present study, we investigated whether selected plant constituents affect HDAC activity or HDAC1 protein status in the human colon carcinoma cell line HT29. The polyphenols (−)-epigallocatechin-3-gallate (EGCG) and genistein (GEN) as well as two oxidative methyleugenol (ME) metabolites were shown to inhibit HDAC activity in intact HT29 cells. Concomitantly, a significant decrease of the HDAC1 protein level was observed after incubation with EGCG and GEN, whereas the investigated ME metabolites did not affect HDAC1 protein status. In conclusion, dietary compounds were found to possess promising HDAC-inhibitory properties, contributing to epigenetic alterations in colon tumor cells, which should be taken into account in further risk/benefit assessments of polyphenols and alkenylbenzenes.

## 1. Introduction

Cancer is one of the most causes of death in industrial countries. Especially the genesis of tumors of the gastrointestinal tract seems to depend on genetic predisposition, environmental factors, and diet [1]. Evidence has been provided that these factors directly influence epigenetic mechanisms associated with cancer development in humans. Epigenetic mechanisms comprise modulation in DNA methylation, histone modification, and noncoding RNA [2]. DNA methylation/demethylation and histone modifications are controlled by specific enzymes, such as DNA methyltransferase (DNMT), histone acetyltransferase (HAT), and histone deacetylase (HDAC) [3, 4]. One of the major posttranslational epigenetic regulations of gene expression is the modulation of histones via acetylation and deacetylation [5]. HDACs belong to the group of zinc-binding metalloenzymes catalyzing the elimination of acetyl groups from histone tails. Deacetylation results in the tighter wrapping of DNA around the histone core leading to chromatin condensation. Based on this cellular event the accessibility of transcription factors and gene expression is decreased. HDACs are involved in several cellular regulation processes such as transcription, cell cycle progressing, gene silencing, cell differentiation, DNA-replication and DNA-damage response [5, 6]. Up to now, 18 human HDAC enzymes are characterized and classified into four classes: class I HDACs share sequence similarity with the yeast Rpd3 deacetylase; they are ubiquitously expressed and are localized in the nucleus. HDACs of class II are homologous to the yeast Hda1 deacetylase and translocate between cytoplasm and nucleus. Class III HDACs are represented by sirtuins, a family of seven HDACs sharing homology with the yeast silent information regulator 2 protein (Sir2). Class IV HDAC11 shares conserved residues with both class I and II HDACs [7–9]. Various tumor types mainly overexpressed class I HDACs associated with drug resistance and poor prognosis [10]. HDAC inhibitors have emerged as a promising class of therapeutic drugs used in the therapy of Alzheimer or Parkinson disease and cancer [11–17]. 

Epidemiological studies indicate that cancer incidence might be significantly modulated by an enhanced dietary intake of polyphenols with fruits and vegetables [18, 19]. Based on the proposed health benefits, polyphenol preparations have gained increasing popularity on the fast expanding market of food supplements. Within the class of polyphenols the major green tea catechin (−)-epigallocatechin-3-gallate (EGCG), the stilbene resveratrol (found in grapes and wine), several soy isoflavones and carboxylic acid derivatives such as chlorogenic acid (CGA) or caffeic acid have been extensively reported to affect HDAC activity, and all are implicated with a reduced cancer risk [2, 20–24]. However, bioactive compounds can possess also adverse health effects. Methyleugenol (ME) belongs to the class of alkenylbenzenes and occurs in different plants such as nutmeg, pimento, lemongrass, tarragon, basil, star anise, and fennel [25–27]. It is also used as a flavoring agent in jellies, baked goods, beverages, sweets such us chewing gums or ice cream, and as a fragrance in cosmetic products [28]. There is a high interest in the safety evaluation of the food constituent ME, since it has been classified as a genotoxic carcinogen by the Scientific Committee on Food (SCF) [29]. 

**Figure 1:**
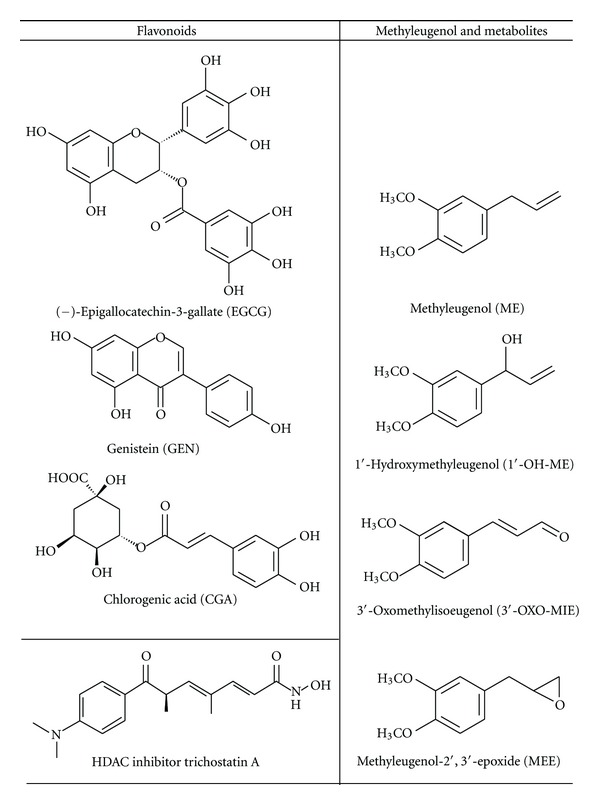
Food-derived polyphenols of different classes and structure of methyleugenol and selected oxidative metabolites.

In this study we addressed the question whether the polyphenols chlorogenic acid (CGA), genistein (GEN), and EGCG (Figure 1) inhibit HDAC1 expression and/or HDAC activity in intact human colon carcinoma cells. Furthermore, we include the genotoxic carcinogen ME and its respective oxidative metabolites 1′-hydroxymethyleugenol (1′-OH-ME), methyleugenol-2′,3′-epoxide (MEE), and 3′-oxomethylisoeugenol (3′-OXO-MIE) (Figure 1) in our testing to get a deeper understanding in mechanisms of action of potential chemopreventive food constituents in comparison to compounds with known adverse health effects.

## 2. Materials and Methods

### 2.1. Chemicals

Dimethyl sulfoxide (DMSO), ME, CGA, and catalase were purchased from Sigma-Aldrich (Steinheim, Germany). GEN and EGCG were purchased from Extrasynthese (Genay Cedex, France). The methyleugenol metabolites 1′-OH-ME, 3′-OXO-MIE, and MEE were synthesized and purified according to the procedures previously described by our lab [30]. Purities of ME metabolites: 1′-OH-ME > 99.5%; MEE > 99.0%; 3′-OXO-MIE > 97.5% (with about 2.0% veratraldehyde), were all checked by 1H-NMR and HPLC. All other chemicals were of the highest or reasonably highest purity commercially available.

### 2.2. Cell Culture

HT29 cells, a human colon adenocarcinoma cell line (Accession number ACC 299, Deutsche Sammlung von Mikroorganismen und Zellkultur (DSMZ), Braunschweig, Germany), were grown in Dulbecco's modified Eagle's medium (DMEM) high glucose (4.5 g/L, Invitrogen Life Technologies, Karlsruhe, Germany). Cell culture medium was supplemented with 10% fetal calf serum (FCS; PAA, Coelbe, Austria) and 1% penicillin/streptomycin (Invitrogen Life Technologies, Karlsruhe, Germany). Cells were cultured at 37°C in a water-saturated atmosphere containing 5% CO_2_. Compounds were dissolved in DMSO and added to the medium to yield a final DMSO concentration of 0.5% (v/v). Control experiments were carried out with medium containing 0.5% of DMSO without test compounds.

### 2.3. Sulforhodamine B Assay

30,000 (24 h), 20,000 (48 h), or 8,000 (72 h) HT29 cells per well were seeded into 24-well plates and allowed to grow for 24 h before treatment. Thereafter, cells were incubated with the respective test compound for 24 h, 48 h, or 72 h in culture medium. Effects on cell growth were determined according to the general method of Skehan et al. [31] modified according to Kern et al. [32]. Incubation was stopped by addition of trichloroacetic acid (50% solution). The fixed cells were stained with a 0.4% solution of sulforhodamine B. The dye was eluted with Tris-buffer (10 mM, pH 10.0) and quantified spectrophotometrically at *λ* = 570 nm. Cell growth was measured as percent survival, determined by the number of treated cells over control cells × 100 (% T/C).

### 2.4. WST-1 Assay

The WST-1 (water soluble tetrazolium) cell proliferation assay was performed according to the manufactures protocol (Roche Diagnostics GmbH, Mannheim, Germany). 5,000 or 3,500 HT29 cells per well, respectively, were seeded into 96-well plates and allowed to grow for 24 h. V79 cells were treated with CGA, EGCG, or GEN for 24 h or 48 h under FCS-containing conditions in the presence of catalase (100 U/mL). In each experiment a solvent control (DMSO 0.5%) and a positive control IGPAL-CA630 (0.5%) were included. After treatment, medium was removed, cells were rinsed with 100 *μ*L/well PBS buffer, and then 100 *μ*L/well serum-free medium was added. 10 *μ*L/well of cell proliferation reagent WST-1 was added to the cells. After 3 h absorbance was measured at *λ* = 450 nm. 

### 2.5. HDAC Enzyme Activity

HDAC enzyme activity was determined using HDAC Assay Kit obtained from Cayman Chemical Company (Ann Arbor, MI, USA) according to the manufactures protocol. 5,000 HT29 cells per well were seeded in 100 *μ*L culture medium in black, clear bottomed 96-well plates. Cells were incubated with CGA, GEN, or EGCG in the presence of catalase to avoid hydrogen peroxide formation, or with ME and its oxidative metabolites at the respective concentrations. After 24 h the plate was centrifuged at 500 ×g for 5 minutes and the culture medium was aspirated. 200 *μ*L of diluted Assay Buffer (kit constituent) was added to each well, the plate was centrifuged at 500 ×g for 5 minutes, and the supernatant was aspirated. Thereafter, 90 *μ*L of culture medium or positive control (recombinant HDAC1) was added to noninhibited sample wells, and 80 *μ*L culture medium with 10 *μ*L trichostatin A was added to the inhibited samples, respectively. The HDAC reaction was initiated by adding 10 *μ*L of the diluted HDAC substrate (BOC-*N* ^*ε*^-acetyl-L-lysine-7-amino-4-methylcoumarin) to each well. The plate was incubated for three hours at 37°C. Cells were lysed by the addition of 50 *μ*L Lysis/Developer Mixture (kit constituent) and the plate was shaken for 2 minutes. After an incubation period for 15 minutes at 37°C fluorescence was measured at an excitation wavelength of *λ*_ex_ = 340–360 nm and an emission wavelength of *λ*_em_ = 440–465 nm. A deacetylated standard (kit constituent) was used to prepare a standard curve for the quantification of HDAC activity.

### 2.6. Western Blot Analysis

4.5 × 10^6^ HT29 cells per Petri dish were seeded in culture medium and allowed to grow for 48 h. Cells were incubated with CGA, GEN, and EGCG in the presence of catalase (see above), or with ME and its oxidative metabolites at respective concentrations. After 24 h of incubation the culture medium was removed and the cells were washed with ice-cold phosphate buffered saline (PBS) for two times and abraded on ice with 200 *μ*L RIPA buffer (65 mM Tris, 154 mM NaCl, 1 mM EDTA, 1% IGEPAL-CA630, pH 7.4). Thereafter the lysate was mixed for 1 minute and was centrifuged for 10 min (20,000 ×g, 4°C). The supernatant was separated by SDS-PAGE (12% polyacrylamide gel) and the proteins were transferred onto a nitrocellulose membrane. Western blot analysis was performed using a goat polyclonal antibody against human HDAC1 (Santa Cruz, Heidelberg, Germany) and an anti-goat IgG-HRP conjugated secondary antibody (Santa Cruz, Heidelberg, Germany). Alpha-tubulin was used as a loading control. The respective chemoluminescent signals (Lumi-GLO, Santa Cruz, Heidelberg, Germany) were analyzed using a LUMI-IMAGER (Roche, Mannheim, Germany). Arbitrary light units were plotted as test over control [%].

## 3. Results and Discussion

### 3.1. Results

#### 3.1.1. Cell Growth Inhibition

**Figure 2:**
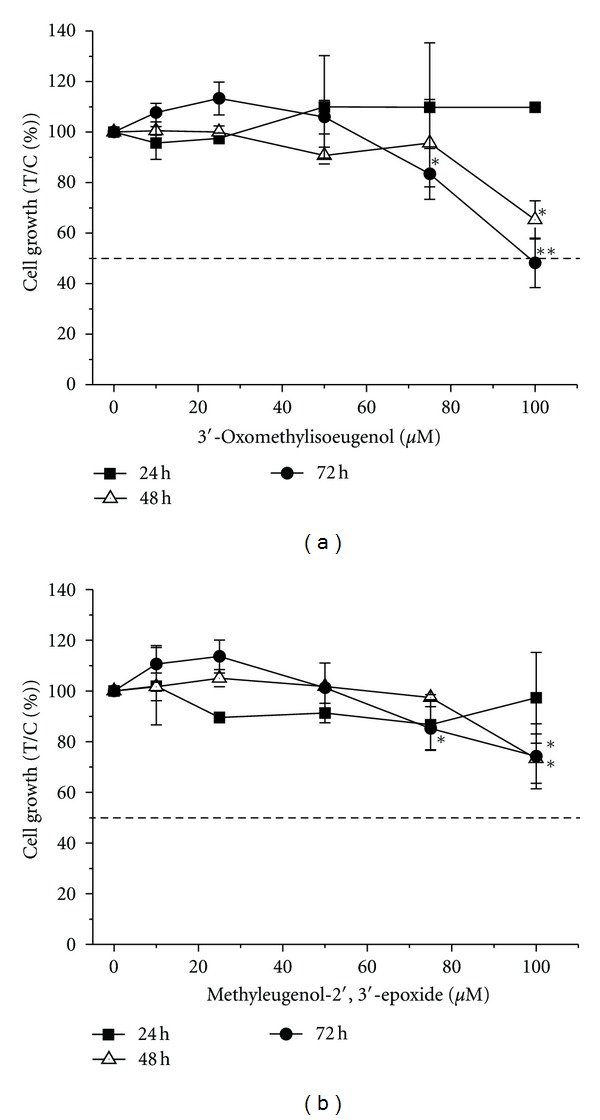
Inhibition of tumor cell growth *in vitro* by (a) 3′-oxomethylisoeugenol (3′-OXO-ME) and (b) methyleugenol-2′,3′-epoxide (MEE). Growth inhibition was determined using the sulforhodamine B assay according to Kern et al. [32]. HT29 cells were incubated with the respective compound for 24–72 h. Growth inhibition was calculated as survival of treated cells over control cells (treated with the vehicle 0.5% DMSO) × 100 [T/C%]. The values given are mean ± SD (standard deviation) of at least four independent experiments, each performed in quadruplicate. The significances indicated were calculated in relation to the solvent control DMSO 0.5% v/v (Student's *t*-test: *P ≤ 0.05; **P ≤ 0.01).

Growth inhibitory properties of methyleugenol and selected methyleugenol metabolites in HT29 cells were determined using the sulforhodamine B (SRB) assay over 72 h. No growth inhibitory properties were observed for ME and 1′-OH-ME using concentrations up to 100 *μ*M and 72 h of incubation (Table 1). Whereas 3′-OXO-MIE reduced cell growth in a time and concentration-dependent manner (Figure 2(a)) with a 50% inhibitory concentration (IC_50_-value) of 100 ± 14 *μ*M (72 h, Table 1). The oxidative metabolite MEE was found to possess significant growth inhibitory properties without reaching an IC_50_-value (Figure 2(b), Table 1). Thus, growth inhibitory potency of the investigated alkenylbenzenes in HT29 cells ranked as follows: 3′-OXO-MIE > MEE > 1′-OH-ME *≈* ME.

Cytotoxicity of CGA, GEN, and EGCG was determined in the presence of catalase (100 U/mL) to avoid hydrogen peroxide formation after an incubation period of 24 h or 48 h using the WST 1 (water soluble tetrazolium salt) assay. CGA, GEN, and EGCG did not exhibit cytotoxic effects up to the highest concentration implemented in our further investigations on HDAC expression and activity (data not shown).

#### 3.1.2. Modulation of HDAC Activity

**Figure 3:**
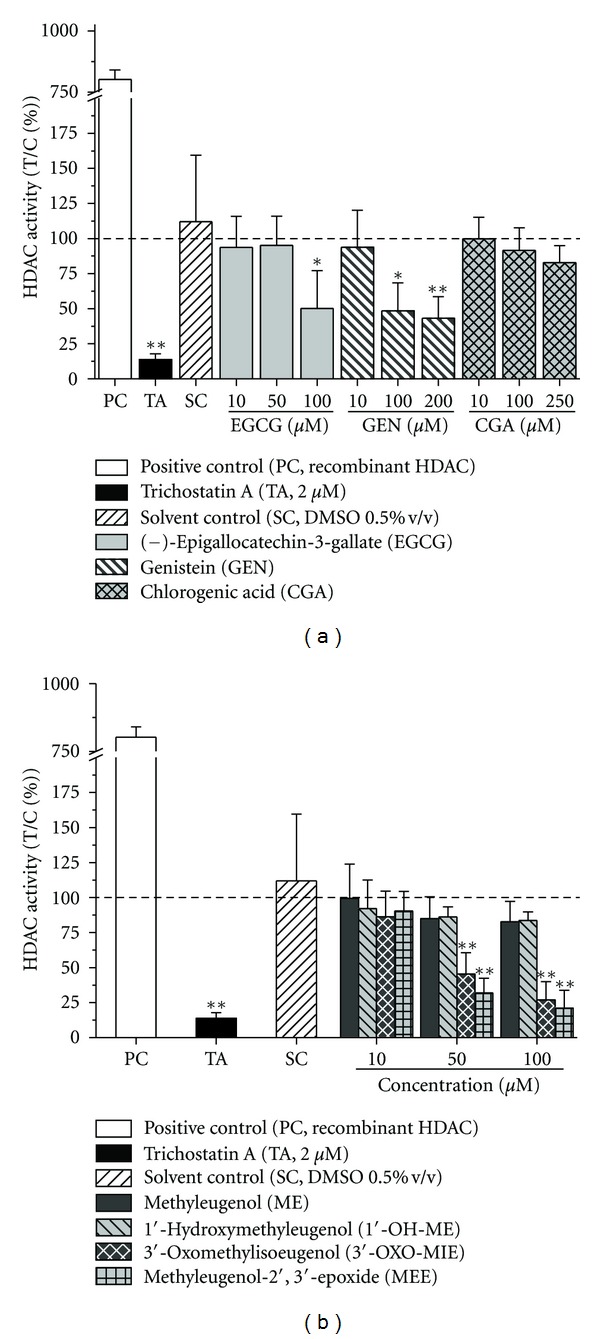
Inhibition of HDAC1 activity in HT29 cells after 24 h of incubation with (a) polyphenols in the presence of catalase (100 U/mL) or (b) methyleugenol and methyleugenol metabolites. HDAC activity was determined by the metabolic rate of a specific HDAC1 substrate. Recombinant HDAC1 was included as positive control (PC) and trichostatin A (TA) as a specific HDAC inhibitor. The data presented are mean ± SD of at least three independent experiments, each performed in a duplicate. The significances indicated were calculated in relation to the solvent control DMSO 0.5% v/v (Student's *t*-test: *P ≤ 0.05; **P ≤ 0.01).

To address the question whether polyphenols or ME and its respective metabolites modulate HDAC activity in intact cells, HT29 cells were incubated with the test compounds for 24 h and enzyme activity was determined in an HDAC cell-based activity assay kit. The specific HDAC inhibitor trichostatin A (Figure 1) was used as a positive control (80% inhibition at 2 *μ*M, Figures 3(a) and 3(b)). EGCG and GEN showed inhibitory effects on cellular HDAC activity in a concentration-dependent manner (Figure 3(a)). The soy isoflavone GEN diminished enzyme activity with an IC_50_-value of 97 ± 18 *μ*M. The green tea catechin EGCG inhibited HDAC activity by about 50% at 100 *μ*M. In contrast, CGA exhibited no statistical significant effect on cellular HDAC activity in HT29 cells up to 250 *μ*M (Figure 3(a)). 

Within the group of alkenylbenzenes ME and 1′-OH-ME did not affect HDAC activity up to 100 *μ*M (Figure 3(b)). However, the ME-derived oxidative metabolites 3′-OXO-MIE and MEE significantly inhibited cellular HDAC activity at concentrations ≥50 *μ*M (Figure 3(b)), reaching the following IC_50_-values: 54 ± 20 *μ*M (3′-OXO-MIE) and 38 ± 3 *μ*M (MEE), respectively.

#### 3.1.3. Effects on HDAC1 Protein Levels

**Figure 4:**
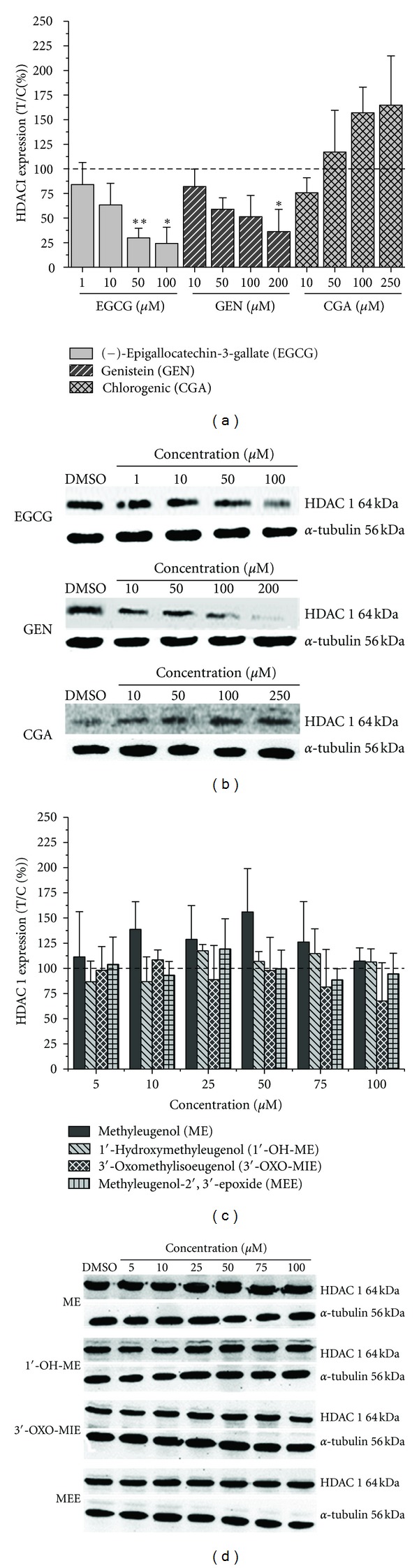
Western blot analysis and representative western blots of HDAC1 expression in HT29 cells after 24 h treatment with polyphenols in the presence of catalase (100 U/mL) ((a) and (b)) or methyleugenol and respective metabolites ((c) and (d)). DMSO: solvent control (0.5% v/v), *α*-tubulin: loading control. Arbitrary light units were plotted as test over control [%], with the respective solvent control (0.5% v/v DMSO) set to 100%. The presented data are mean ± SD of four independent experiments with similar outcome. The significances indicated were calculated in relation to the lowest applied concentration (Student's *t*-test: *P ≤ 0.05; **P ≤ 0.01).

We further addressed the question whether the modulation of HDAC activity by secondary plant constituents in HT29 cells is associated with changes in the amount of HDAC protein. The impact of the test compounds on HDAC1 protein status in HT29 cells after 24 h of incubation was detected by western blot analysis using a goat polyclonal antibody against human HDAC1. Incubation of HT29 cells with CGA increased the HDAC1 protein level up to 250 *μ*M without statistical relevance (Figures 4(a) and 4(b)). In contrast, the incubation of HT29 cells with GEN resulted in a significant decrease of HDAC1 protein at a concentration of 200 *μ*M (Figure 4(a)). EGCG also decreased the protein level of HDAC1 in a concentration-dependent manner. The HDAC1 status was significantly reduced at concentrations ≥50 *μ*M (Figure 4(a)).

After treatment of HT29 cells with methyleugenol or the selected metabolites, no changes in the amount of HDAC1 protein were detected by western blot analysis (Figures 4(c) and 4(d)). 

### 3.2. Discussion

Dietary polyphenols have been demonstrated to exhibit cancer preventive and cancer therapeutic activity [18, 19, 34]. The impact of polyphenols on enzymes involved in cell cycle regulation, apoptosis, invasion, metastasis, and angiogenesis has been associated with their beneficial health effect. More recently, polyphenols have been found to alter gene expression by posttranslational modifications, including DNA methylation or histone acetylation/deacetylation [8, 20, 35, 36]. In the present study, EGCG, the most abundant catechin in green tea, was shown to inhibit HDAC activity and HDAC1 protein expression in human colon carcinoma HT29 cells after 24 h of incubation (Figures 3(a) and 4(a)). Green tea polyphenols and notably EGCG have been reported to diminish HDAC activity, mRNA expression of HDAC1 and protein expression of class I HDACs in prostate cancer cells [20, 21]. The treatment of LNCaP human prostate cancer cells with EGCG (5–20 *μ*M) resulted in a dose- and time-dependent decrease of class I HDACs (HDAC1, 2, 3, and 8) [21]. These results are in line with our findings that incubation with EGCG (≥50 *μ*M) significantly diminished the protein level of HDAC1 in the human carcinoma cell line HT29 (Figures 4(a) and 4(b)), whereas a significant reduction of HDAC activity was also determined but at higher concentrations (100 *μ*M, Figure 3(a)). The soy isoflavone GEN significantly inhibited HDAC activity at concentrations ≥100 *μ*M (Figure 3(a)). HDAC1 protein expression was significantly reduced after incubation with 200 *μ*M GEN (Figures 4(a) and 4(b)). Therefore it can be assumed that other HDAC isoforms are affected by GEN leading to the more pronounced reduction of HDAC activity which needs further investigations.GEN and several other isoflavones have been recently described as HAT activators and HDAC inhibitors in esophageal, prostate, breast, and renal tumor cancer cell lines [24, 37–40]. HDAC activity was slightly decreased after the treatment with CGA (250 *μ*M),whereas the protein level of HDAC1 was shown to be moderately increased, but both effects were without statistical relevance. HDAC activity of a HeLa nuclear extract was inhibited by CGA with an IC_50_-value of 375 *μ*M [23]. To our knowledge, modulation of HADC activity by CGA in cellular systems has not been reported so far. CGA is one major polyphenol of our daily diet, which occurs in several food sources such as apples, potatoes, or coffee [41]. Coffee consumers ingest about 1 g/d CGA, whereas a daily intake of chlorogenic acid <0.1 g/day has been reported for individuals who do not drink coffee [41, 42]. The average daily intake of soy isoflavones in the western diet has been estimated of about 1–3 mg/day, 30–50 times lower than in Asian countries [43]. A cup of green tea (200 mL) contains about 150 mg EGCG mainly consumed in Asian countries [44]. Several studies have demonstrated that only a small percentage of ingested GEN, CGA, or EGCG appears in the blood with mean peak plasma concentration levels of nanomolar concentrations [45–47]. Most of the published *in vitro* investigations, including our study, used the respective polyphenols at micromolar concentrations [20–24]. The rather poor bioavailability of polyphenols needs to be considered when we extrapolate results obtained *in vitro* to situations *in vivo*. 

Furthermore, it has to be mentioned that cytotoxic effects of the polyphenols EGCG, GEN, and CGA in the implemented concentration range has been intensively discussed [32, 33, 48, 49]. Different authors have reported a substantial hydrogen peroxide formation under cell culture conditions, resulting from the reaction of the used polyphenols with yet unknown culture media constituents, which influences the cellular effectiveness of the respective polyphenols [50–55]. EGCG exhibited potent growth inhibitory properties, yet in our experiments in accordance with earlier results, the presence of catalase significantly attenuated this effect [55]. The addition of catalase resulted in a loss of any cytotoxic effects of EGCG up to 100 *μ*M (data not shown), suggesting that the formation of hydrogen peroxide acts as a major contributor of the reported cytotoxicity. In contrast, no details on hydrogen peroxide formation after cell treatment with GEN have been reported so far. GEN, in the absence of catalase, reduced dose-dependently (0–100 *μ*M) the cell viability of HT29 cells after 72 h of incubation [33], whereas in our experiments no cytotoxicity was measured up to 200 *μ*M (24 h) neither in presence nor in absence of catalase (data not shown).

Taken together, a hydrogen peroxide-scavenging system to *in vitro* cell culture assays using plant polyphenols seems to be needed. By the use of catalase (100 U/mL) any artificial contribution of hydrogen peroxide to the inhibitory effects on HDAC1 protein status and HDAC activity can be excluded.

Within the class of alkenylbenzenes only the two oxidative metabolites 3′-OXO-MIE and MEE observed significant growth inhibitory properties on HT29 cells (Figure 2, Table 1) according to previous results of our group on V79 Chinese hamster lung fibroblasts cells [56]. With respect to the metabolic activity of HT29 cells in contrast to V79 cells we have postulated a variably sensitivity against the test compounds, which was not the case. Furthermore, six oxidative ME-metabolites, including the here-investigated ones, have been reported to reduce cell viability in metabolic competent primary rat hepatocytes by using two different assays. The authors found the highest cytotoxicity for the main ME-metabolite 1′-hydroxymethyleugenol and the secondary metabolite 1′-oxomethyleugenol, whereas ME was found to be much less cytotoxic [57]. These results are in accordance with the studies of Burkey et al. [58], where ME only exhibited marginal cytotoxicity in rat hepatocytes at concentrations up to 3 mM.

The levels of HDAC activity within cells can be altered via direct inhibition of the HDAC enzyme and changes in HDAC protein levels. In the study presented here, the metabolites 3′-OXO-MIE and MEE were firstly reported to potently diminish HDAC enzyme activity (Figure 3(b)) without modulating the protein status of HDAC1. Further work will be needed to investigate whether the metabolites directly suppress HDAC activity or whether protein expression of the other HDAC isoforms will be suppressed. 

HDAC inhibition leads to genomic instability by a variety of mechanisms. This effect may contribute to the cytotoxicity of these drugs. Furthermore, HDAC inhibitors sensitize DNA to exogenous genotoxic damage and induce the generation of reactive oxygen species. At least, HDAC inhibitors could induce chromosome missegregation [59]. Some dietary constituents have been reported to enhance DNA damage and to affect HDAC activity in cancer cells. The glucosinolate sulforaphane induced DNA double strand breaks in the human colon carcinoma cell line HCT116 [8]. Furthermore, HDAC3 and HDAC6 protein expression was decreased by sulforaphane in a time-dependent manner, leading to acetylation of histone H4 and tubulin, respectively [60]. Within the class of polyphenols EGCG, GEN, and quercetin, a flavonoid found in foods such as citrus fruit, apples, and onions, or resveratrol, has been characterized as HDAC inhibitors with DNA-damaging properties recently reviewed by [8]. We reported previously that 3′-OXO-MIE and MEE significantly induced DNA strand breaks [56]. However, the underlying mechanism of action has been not identified so far. Additional studies are required to elucidate whether downregulation of DNA repair or cell cycle regulating events may be involved in the DNA damaging mechanisms of 3′-OXO-MIE and MEE.

## 4. Conclusion

The present study shows that the polyphenols EGCG and GEN and the two ME metabolites 3′-OXO-MIE and MEE potently diminished the activity of HDAC in intact colon carcinoma cells. We further demonstrated that modulation of HDAC activity is associated with the suppression of HDAC1 protein status by polyphenols, whereas the ME metabolites did not affect the protein level of HDAC1. These results illustrate an interference of EGCG and GEN with epigenetic pathways which may contribute to the idea that dietary polyphenols have potentially chemopreventive effects. Furthermore, we show that via bioactivation of the prominent food carcinogen ME, metabolites not only with potential genotoxic, but also with HDAC inhibitory properties will be generated which may contribute to their DNA-damaging properties. In summary, the results presented reveal that more investigations on the mechanism of action for future risk/benefit assessment of polyphenols and alkenylbenzenes will be necessary.

## Figures and Tables

**Table 1:** Cytotoxic properties of the test compounds in HT29 cells.

Compound	IC_50_-value [*μ*M]		
	24 h	48 h	72 h
EGCG	n.d.^a, b^	n.d.^a, b^	40 ± 6^c^
GEN	n.d.^a, b^	>100^b^	~50^d^
CGA	n.d.^a, b^	n.d.^a, b^	205 ± 53^c^
ME	n.d.^a^	n.d.^a^	n.d.^a^
1′-OH-ME	n.d.^a^	n.d.^a^	n.d.^a^
3′-OXO-MIE	n.d.^a^	>100	100 ± 14
MEE	n.d.^a^	>100	>100

^a^No cytotoxicity up to the highest concentration implemented in the testing; ^b^data was performed in the presence of catalase (100 U/mL) to avoid hydrogen peroxide formation; ^c^without catalase; previously reported by Kern et al. [32]; ^d^previously reported by Yu et al. [33].
